# New Origins of Yeast, Plant and Bacterial-Derived Extracellular Vesicles to Expand and Advance Compound Delivery

**DOI:** 10.3390/ijms25137151

**Published:** 2024-06-28

**Authors:** María Fernández-Rhodes, Cristina Lorca, Julia Lisa, Iolanda Batalla, Alfredo Ramos-Miguel, Xavier Gallart-Palau, Aida Serra

**Affiliations:** 1 +Pec Proteomics Research Group (+PPRG)—Neuroscience Area, Biomedical Research Institute of Lleida Dr. Pifarré Foundation (IRBLLEIDA)—University Hospital Arnau de Vilanova (HUAV), 80 Av. Rovira Roure, 25198 Lleida, Spain; mfernandezr@ibecbarcelona.eu (M.F.-R.); cristinalorca92@gmail.com (C.L.); jlm13@alumnes.udl.cat (J.L.); 2 Department of Medical Basic Sciences, University of Lleida (UdL), 25198 Lleida, Spain; 3 Institute for Bioengineering of Catalonia (IBEC), C. Baldiri Reixac, 10-12, 08028 Barcelona, Spain; 4 Psychiatry Unit, Hospital Universitari Santa Maria, Medicine Department, Universitat de Lleida (UdL), 25198 Lleida, Spain; ibatalla@gss.cat; 5 Department of Pharmacology, University of the Basque Country UPV/EHU, 48940 Leioa, Spain; alfredo.ramos@ehu.eus; 6 Biocruces Bizkaia Health Research Institute, 48903 Barakaldo, Spain; 7 Centro de Investigación Biomédica en Red en Salud Mental CIBERSAM, Instituto de Salud Carlos III, 48940 Leioa, Spain

**Keywords:** extracellular vesicles, food industry by-products, drug loading, editing, drug delivery

## Abstract

Extracellular vesicles (EVs) constitute a sophisticated molecular exchange mechanism highly regarded for their potential as a next-generation platform for compound delivery. However, identifying sustainable and biologically safe sources of EVs remains a challenge. This work explores the emergence of novel sources of plant and bacterial-based EVs, such as those obtained from food industry by-products, known as BP-EVs, and their potential to be used as safer and biocompatible nanocarriers, addressing some of the current challenges of the field. These novel sources exhibit remarkable oral bioavailability and biodistribution, with minimal cytotoxicity and a selective targeting capacity toward the central nervous system, liver, and skeletal tissues. Additionally, we review the ease of editing these recently uncovered nanocarrier-oriented vesicles using common EV editing methods, examining the cargo-loading processes applicable to these sources, which involve both passive and active functionalization methods. While the primary focus of these novel sources of endogenous EVs is on molecule delivery to the central nervous system and skeletal tissue based on their systemic target preference, their use, as reviewed here, extends beyond these key applications within the biotechnological and biomedical fields.

## 1. Introduction

Extracellular vesicles (EVs) are a highly regarded mechanism of intercellular communication due to their ability to offer mechanistic insights into pathogenic processes, provide potential biomarkers with prognostic and diagnostic capabilities, and serve as a promising next-generation delivery platform for compound administration [1,2,3,4]. These spherical vesicles are released and internalized by a diverse array of cell types across different biological domains, serving as crucial mediators of molecular exchange between cells [5]. Given their unique characteristics, EVs are hypothesized to play essential roles in both promoting healthy statuses and contributing to disease [6]. Additionally, these have been recognized as promising means for transporting medications and bioactive substances, resulting in excellent prospective applications as nanocarriers [7].

These vesicles exhibit advantageous characteristics as nanocarriers, including low immunogenic reactions, the ability to traverse systemic tight junctions and promote targeted organ delivery, though, their optimal utilization in the field still encounters significant challenges [7]. Safety concerns, scalability issues, and the identification of compatible physicochemical attributes notably hinder their widespread application [8]. These challenges are partly rooted in the prevalent use of immortalized cell lines as primary sources of EVs for research purposes, raising apprehensions involving human toxicity and source scalability [9]. Similarly, the advance and implementation of laboratory-synthesized EVs and liposomes as potential nanocarriers are constrained by the challenges associated with the current commonly existing EVs sources [10].

The utilization of sustainable, eco-friendly sources for autologous EVs, particularly those derived from waste products of the food industry, referred to as BP-EVs, presents significant advantages. Compared to traditional sources, BP-EVs exhibit remarkable non-toxicity, closely resembling the vesicles found in commonly consumed foods. They demonstrate exceptional oral and intravenous bioavailability and possess a remarkable ability to target specific organs, particularly the central nervous system (CNS), the skeletal tissues, and the liver. Furthermore, BP-EVs can be conveniently functionalized using common functionalization methods [11]. In summary, we believe that novel sources of EV-oriented nanocarriers based on plant, bacterial, and yeast, as reviewed here, can have a profound impact on the current expanding bioeconomy and biomedicine sectors that require special attention.

## 2. Extracellular Vesicles

**Figure 1:**
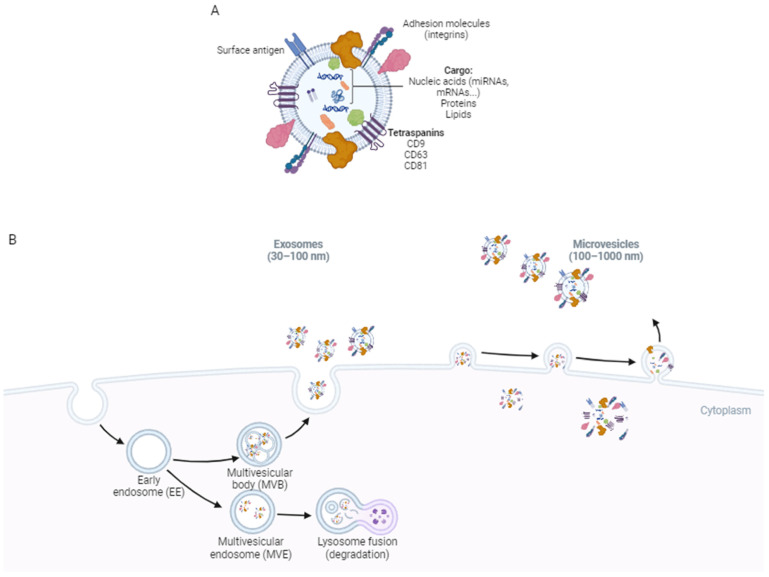
EV characteristics and biogenesis pathway. (**A**) Graphical representation of the composition and structure of an EV (**B**) Biogenesis pathway. Various components and cargoes destined for EVs are distributed throughout the cytoplasm and membranes. Specific elements and cargoes earmarked for future EVs are gathered and recruited. The recruited elements aid in membrane bending and invagination. Membrane scission occurs, resulting in the formation of either microvesicles or intraluminal vesicles. The multivesicular body (MVB) is transported to the plasma membrane. The MVB fuses with the plasma membrane, liberating the intraluminal vesicles (now known as exosomes) into the extracellular space. Figure created using the tool at https://www.biorender.com/, accessed on 15 March 2024.

EVs are characterized by a wide variety of spherical particles, which are nanoscale in size and have diameters ranging from 20 to more than 2500 nanometers. These particles are derived from cells and are enclosed within a phospholipid (PL) bilayer membrane [12]. These non-replicative particles are molecularly composed of lipids, proteins, and nucleic acids and serve as miniature capsules that can displace, protect, and deliver various cargoes [12]. EVs were traditionally thought of as mere cellular debris and were often referred to as “cellular dust” [13]. Despite this, they have been shown to play crucial roles and possess remarkable properties as biomarkers for human diseases, although they were historically disregarded in these senses for many years [13]. State-of-the-art technologies recognize that EVs are generated by all types of cells and are present in various biological fluids. The International Society for Extracellular Vesicles (ISEV) has officially recognized that extracellular vesicles possess specific characteristics, such as their lipid-based membranous bilayer structure, their natural release and facilitated endocytosis at the cellular level, and their inability to replicate independently. Per the ISEV guidelines, EVs are classified based on their natural development pathways, which divides them into three distinct categories: exosomes, microvesicles, and apoptotic bodies. However, the latest classification focuses on categorizing them based on their size distribution as small EVs and larger EVs, as many of their molecular features are shared among all vesicles within the distribution [14] (Figure 1).

EVs have emerged as a robust means of communication, enabling signaling between alike and diverse living beings. They serve as carriers for molecular messages, facilitating communication among cells whether they are nearby or distant. Serving as mediators across all four levels of communication, EVs play a highly important role [12]. These vesicles demonstrate a distinctive capacity to enclose and protect diverse messenger molecules, encompassing both hydrophilic and hydrophobic substances. Additionally, they exhibit complex compositions that provide direct insights derived from the originating cell [15]. Additionally, studies have shown that specific EVs exhibit a preference for particular cell types or tissues [16]. This groundbreaking find has revealed a previously unexplored and complex research area with multiple sub-branches, endowing these particles with promising potential as tools for enhancing our understanding of cellular communication and potentially intervening in it. Moreover, recent findings indicate that EVs could emerge as the foremost contenders for utilization as personalized and customizable targeted nanovectors in health in the foreseeable future [16].

### 2.1. Diversity of EVs

#### 2.1.1. EVs Originated from Fungi and Yeast

Despite being initially identified in the early 1970s, it was not until 2007 that fungus- and yeast-originated extracellular vesicles (FY-EVs) were comprehensively analyzed, with particular attention given to the yeast species *Cryptococcus neoformans* [17]. In the same way that bacteria and plants possess sturdy cell walls, fungi cells are also safeguarded by a similar structure composed mainly of glycoproteins and intricate carbohydrates. Additionally, fungi have a unique polysaccharide known as chitin [18]. Initially, these structures were perceived as obstacles impeding vesicular transport, due to their inflexibility and scarcity of large pores. However, it is now understood that fungi cell walls are adaptable features capable of rearrangement during cellular division or EV biogenesis. In recent years, extensive research has been conducted to understand the primary functions of FY-EVs and their involvement in a variety of biological processes, including the reconstitution of cellular membranes [19] and biological film generation [20], and host interactions [21] have been identified, with clear evidence demonstrating their capacity to influence recipient cell behavior [22]. The fungus production of EVs has been observed in various species, such as the widely recognized yeast *Saccharomyces cerevisiae*, which is commonly used in brewing, and *Candida albicans*, a prevalent microorganism in the human microbiome. Additionally, filament-linked species such as *Aspergillus fumigatus* and *A. flavus*, causative agents of invasive aspergillosis, have also been shown to produce EVs [23].

Indeed, FY-EVs and mammalian EVs exhibit analogous categorization, as they both formulate in exosome and microvesicle-like entities. Although the exact mechanisms responsible for the production of FY-EVs have not been completely understood, there are indications of similarities in biogenesis pathways with mammalian vesicles, suggesting highly evolutionarily conserved mechanisms [24]. The production of EVs involves various cellular processes, one of which is the endosomal pathway. Evidence suggests that both fungi and mammals engage in this process. In fungi, for example, periplasmic vesicles (PVs) have been observed within the cell walls and the interiors of cells. However, the exact nature of these structures in relation to FY-EVs that have been observed in external environments remains unclear, as it is currently unknown whether they are structurally and functionally equivalent [25]. Further investigation is thus warranted to delve into the intricacies of FY-EV biology, despite the present comprehension.

#### 2.1.2. EVs Originated from Plants

The discovery of plant-derived extracellular vesicles (P-EVs) dates back to the 1960s [26], but they continue to be relatively understudied, and the cellular mechanisms underlying their generation remain largely unknown. The widely accepted notion is that the generation pathways of EVs in plant cells involve the interaction of multivesicular bodies (MVBs) with cellular membranes, leading to the formation of exosomes. In addition, the budding of microvesicles or apoptotic bodies from the cell membrane is also expected. Furthermore, exocyst-positive organelle (EXPO)-mediated secretion is thought to result in the generation of EXPO-positive vesicles [26]. In addition, the mechanisms facilitating the transit of P-EVs across the cell wall and their release into the extracellular space, as well as the diverse secretion pathways involved, are not yet well understood. Consequently, the categorization of P-EVs remains in its preliminary stages. P-EVs can be classified based on their association with specific markers that are indicative of their biogenesis mechanisms, leading to the identification of at least three distinct subcategories: Tetraspanin (TET)-positive exosomes, which are characterized by the presence of TET-like proteins and originate from multivesicular bodies (MVBs); EVs derived from the EXPO (EXPO-derived EVs); and PEN1-positive EVs, which contain the penetration 1 protein and are implicated in stress responses, although the origin of these EVs remains unidentified [27]. P-EVs play a crucial role in vital cellular processes, particularly in safeguarding cells against pathogens, much like in other biological contexts [27]. Nevertheless, despite the existing body of knowledge and the emphasis placed on this matter repeatedly, further research is necessary to enhance our understanding of P-EVs and the functional implications they possess, as similarly stated above regarding FY-EVs.

#### 2.1.3. EVs Originated from Bacteria

Bacterial EVs (B-EVs) typically have diameters ranging between 20 and 400 nanometers and are classified based on their source, structure, and chemical composition. The primary distinction between B-EVs produced by Gram-negative and Gram-positive bacteria lies in their unique characteristics, which are evident in the structures of the vesicles themselves [28].

##### Gram-Negative Bacteria-Derived EVs

Gram-negative bacteria are characterized by the presence of two membranes, an outer layer that includes lipopolysaccharide (LPS) on its surface and an inner (or cytoplasmic) layer that is separated from the peptidoglycan periplasmic space [29]. The outer membrane vesicles (OMVs) of Gram-negative bacteria, measuring between 50 and 250 nanometers in size, represent the most common type of EVs found in these organisms. OMVs are formed directly from the outer membrane and are encased within the LPS layer, which contains outer membrane proteins, periplasmic components, and a specific lipid composition [30]. Although OMVs can carry cytoplasmic molecules, the processes that control the cargo loading and sorting of these vesicles are not yet fully understood [29]. Compared to conventional EVs, outer–inner membrane vesicles possess a distinct characteristic in having double bi-layered membranes that correspond to the outer and inner membranes found in Gram-negative bacteria. Similarly to OMVs, the outer membrane of these vesicles is abundant in LPS, while peptidoglycan can be found in the periplasmic space [28].

##### Gram-Positive Bacteria-Derived EVs

Gram-positive bacteria differ from Gram-negative bacteria in the presence of a thick layer of peptidoglycan surrounding their plasma membrane, which functions as a barrier for B-EVs attempting to exit the cell. On the other hand, Gram-negative bacteria lack this additional layer of protection [31]. Therefore, the term “cytoplasmic membrane vesicle” (CMV) is proposed for the B-EVs secreted by Gram-positive bacteria. This term is applicable to both B-EVs produced by the bacteria themselves and those released from apoptotic cells. Regardless of the source, endolysin triggers the formation of CMVs, which then bud into the extracellular space and circumvent the peptidoglycan barrier [28]. B-EVs play a crucial role in delivering virulence factors, nucleic acids, and defensive agents to hosts [28]. In particular, they play a significant role in the ecosystem, especially in marine environments, where they actively contribute to the progression of the circular carbon cycle [32].

## 3. EVs Used as Nanovectors

The effectiveness of compound delivery is commonly hindered by their instability and their limited capacity to reach target tissues in a majority of cases where compounds are administered in their free form. To surmount these obstacles, carriers are frequently utilized for the purpose of efficient drug delivery. Drug delivery systems encompass a variety of technologies that encapsulate and transport drugs through circulation and cell/tissue barriers, thus overcoming pharmacokinetic challenges and enhancing the potency of their effects [7]. In recent times, extensive research has been conducted to identify effective delivery systems for targeted drug administration. The development of synthetic carriers, such as liposomes, microspheres, and polymeric nanoparticles, has been a focal point due to their potential to deliver a variety of compounds, including small molecules, peptides/proteins, DNA/RNAs, and antibodies [33]. However, the use of artificial transporters is not without its drawbacks, as they have a tendency to evoke a reactive immune response upon recognition as foreign entities [33].

EVs have emerged as valuable nanovector resources in healthcare due to their capacity to transport proteins, lipids, and nucleic acids with precision in an encapsulated manner to specific organs and cells. These vesicles exhibit distinctive features and offer several advantages over artificial nanocarriers. Some of these advantages include efficient cellular uptake, remarkable stability, compatibility with biological systems, minimal immune reactions, the ability to traverse biological barriers, such as the blood–brain barrier (BBB), secure cargo transport, and the potential for the targeted delivery of biologically relevant molecules [34]. As such, EVs have the potential to significantly enhance their pharmacological effects reducing toxicity. Furthermore, EVs hold promise for diverse applications including immunotherapy, gene therapy, tissue engineering, and vaccine development [35]. One possible application of these materials is in the delivery of compounds able to promote the repairing of tissues [36]. Therefore, devising appropriate strategies for utilizing EVs as nanocarriers could pave the way for their application across a broad spectrum of medical endeavors [37].

### 3.1. Native EVs: Nanovectors Outperforming Lab-Generated Nanocarriers

Lab-generated liposomal vesicles have been extensively investigated for their potential as nanotransporters [38]. Compared to EVs, synthetic nanoparticles show a simpler structure and offer appealing properties such as higher-scale production, cost- and time-effectiveness, and simpler standardization protocols [39]. However, when used as stimuli, these synthetic particles find inherent challenges that hinder their efficiency as nanocarriers [38]. One of the principal problems is their lack of biocompatibility. Engineered materials may elicit cytotoxicity and adverse immune responses, restricting their suitability for biomedical applications [40]. In addition, liposomal vesicles frequently face problems with accumulation when produced in laboratory settings, which could lead to negative consequences in the long run [41]. Furthermore, there are ongoing difficulties connected to lab-generated nanovectors, particularly in terms of their precision in targeting and their capacity to overcome biological obstacles such as the BBB. These limitations restrict their effectiveness in delivering drugs to specific body regions, particularly in the brain [42].

In contrast to lab-generated nanovectors, endogenous or native cell-generated EVs, have inherent biocompatibility, low immunogenicity [43], and reduced toxic effects. Native EV carriers are distinguishable by recipient cells and possess a larger capacity of circulation than lab-generated alternatives [44]. These carriers have the ability to connect with specific cell types, which enables focal delivery, in turn limiting off-target issues and ultimately enhancing treatment effectiveness [45]. Furthermore, EVs show a higher capacity to surpass biological obstacles, such as the BBB [46], facilitating CNS cargo delivery for specific brain therapies. This feature makes them particularly attractive as treatments regarding brain-related diseases and disorders. However, the large-scale production of EVs remains a challenge [47,48]. Additionally, safety concerns have been raised regarding the use of cancerous cell-derived EVs, as they may possess tumorigenic properties. Furthermore, there are concerns about the reliability of immortalized cell cultures as sources of EVs. Standardizing isolation protocols for these particles is also a complex task due to their high heterogeneity [47]. Despite their potential, thus, there are still several drawbacks to be addressed before EVs can be fully realized as a superior option for biomedical nanoparticles [49].

### 3.2. Use of EVs as Nanocarriers and Facing Challenges

Due to the nanovector capacity of EVs, there is growing interest in their potential use as substance or pharmacological transporters, particularly for chemotherapeutics [50]. However, multiple obstacles hinder their clinical application [51]. Challenges that include obtaining sufficient yield, isolation, storage, the standardization of procedures, EV characterization, safety, loading, and targeting modification are major hurdles impeding the application of EVs as nanocarriers in clinical settings [52]. Immortalized cell lines correspond to some of the most common sources for EV production as therapeutic nanocarriers. These include specifically cultured cells grown in 2D cultures [53]. Nevertheless, they may generate them in limited amounts [54], and transitioning these settings to suspension growth is non-trivial, which hampers their applicability in novel bioeconomy sectors.

The issue of using cell lines to generate EVs presents another concern, particularly with regard to potential safety concerns. Specifically, it is a matter of concern when EVs are derived from immortalized cells, as they could potentially exhibit pro-tumorigenic abilities [55]. Additionally, for EVs to work as nanovectors, the process of loading these vesicles with the appropriate molecule, drug, or cargo is of paramount importance. This requires the employment of suitable techniques, which can be tailored based on the molecular properties of the component in question [55]. Lastly, depending on this, modifications on the EV surface may be required to obtain an optimal targeting and biodistribution while maintaining their low immunogenicity and stability [56]. Even with obstacles in place, the expanding potential of exosomes is evident across academic and commercial domains. These sectors offer tools and resources for isolating, purifying, characterizing, and modifying exosomes, as well as supporting preclinical and clinical trial endeavors [57].

## 4. Bacterial, Yeast, and Plant EVs as Nanocarriers in Biomedicine and Biotechnology: The Case of BP-EVs

BP-EVs are proprietary assets that consist of EVs generated from fermented food industry by-products that are protected by an international patent (PCT/EP2022/080507). These by-products are derived from the production or processing of various food items, such as kefir and other fermented dairy products, as well as plant-based foods like beer and wine, among others [11,58]. The EVs obtained from these by-products range between 30 and 950 nm in diameter, predominantly below 200 nm (50% of the isolates), indicating small-EV enrichment. Furthermore, BP-EVs exhibit comparable metabolome and proteome profiles to those of food-derived EVs, demonstrating no cytotoxic effects [11]. BP-EVs have exceptional oral bioavailability, which is comparable to intravenous administration, and exhibit excellent biodistribution. These vesicles contain exosome markers and show unique in vivo targeting, particularly toward the CNS, liver, and skeletal tissues. The most effective method for obtaining BP-EVs involves an initial centrifugation step to separate cells and insoluble debris from the BP solution, followed by a series of washing and filtration steps to eliminate soluble components from the source material and concentrate the vesicles, a method that is industrially scalable through tangential filtration [11].

In terms of generating biocompatible and safe EVs in sufficient quantities as nanocarriers, FFBPs have been demonstrated by BP-EVs to be a cost-effective and viable alternative to the challenges faced [11]. The integration of industrial scale-up techniques with other industrial and filtration technologies offers a novel and practical solution to overcome some of the major obstacles in EVs research and their utilization as nanovectors [11].

### 4.1. BP-EVs: Industrial Circular Actions with Health Impact

Food waste is a major environmental problem and entails a substantial financial burden for its management. The integration of circular economy principles into the development of BP-EVs contributes to the upcycling of industrial waste. In using FFBPs as a sustainable and biocompatible source for EVs, a novel and environmentally conscious approach has been established. This innovative method transforms potentially discarded food industry waste into advanced nanocarrier tools, creating a highly valuable biomedical resource. Additionally, almost 95% of the residue can be sold for animal feeding after the extraction of BP-EVs. This approach not only addresses industrial waste challenges, but also unlocks opportunities for EV research and biotechnological and biomedical applications.

### 4.2. BP-EVs and Their Use as Nanovectors for Compound Delivery

BP-EVs possess the innate capability to cross the BBB, which makes them a promising candidate for the delivery of drugs targeting the CNS. The market for CNS-focused treatments is substantial, estimated to be worth $612 million globally in 2022 and projected to increase at a constant annual growth rate (CAGR) of 8.9% to reach $938 million by 2027. An example that demonstrates a possible use for BP-EVs is the treatment of glioblastoma multiforme, which is the most prevalent and deadly type of brain cancer in adults and has a 5-year relative survival rate of 39.4% as of 2021 [59]. However, the potential benefits extend beyond glioblastoma to encompass the treatment of neurodegenerative diseases, psychiatric disorders, and some CNS cancers. The utilization of these advanced nanocarriers holds the promise of substantially improving therapeutic outcomes in these challenging conditions.

Additionally, BP-EVs offer a potential solution to the challenge of delivering therapeutics specifically to bone tissue, given their preferential accumulation in this tissue type. This is particularly important due to the limited blood supply to bone tissue, which often requires high doses of therapeutics to achieve effective results. However, this can lead to unwanted side effects due to untargeted delivery. One potential application of BP-EVs is in the treatment of osteoporosis, a metabolic disorder that weakens bone structure, increasing the risk of fractures and resulting in significant morbidity. The market for osteoporosis treatments was valued at $14 billion in 2022, with a projected CAGR of 3.8%. It is important to note [60,61], however, that while BP-EVs exhibit liver targeting capacity, their presence in this organ may primarily be associated with detoxification and excretion. Further research is needed to investigate the potential of BP-EVs in addressing additional health conditions, particularly those affecting the liver [62].

## 5. Edition of EVs to Be Used as Nanocarriers

**Figure 2:**
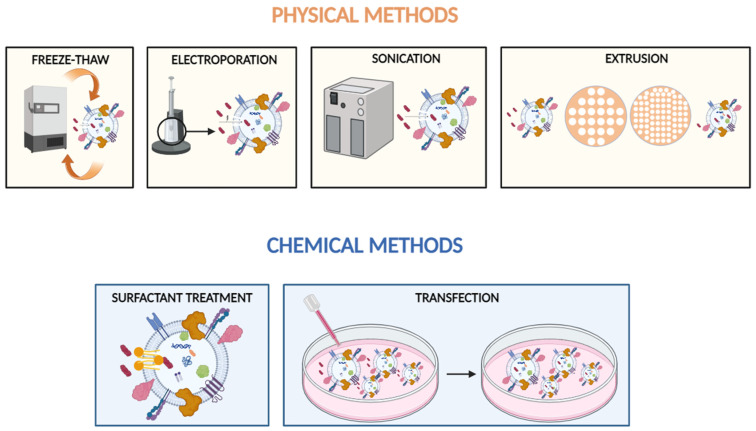
Summary diagram of current available edition methods. Figure created using the tool at https://www.biorender.com/, accessed on 15 March 2024.

Various editing techniques are employed in the engineering of BP-EVs (biomimetic or bioengineered extracellular vesicles) and EVs in general, converting them into nanocarriers capable of modifying their properties to achieve desired therapeutic effects. These methodologies facilitate the customization of EVs to encapsulate molecules both internally and externally, thereby avoiding clearance by the immune, hepatic, or renal systems. Additionally, these methodologies can be employed to exhibit tropism toward specific microenvironments, such as low-pH conditions, and to selectively target particular cells or organs. Furthermore, these methodologies can enhance intracellular cargo delivery and trigger specific cellular responses, among other objectives. Methods of editing can be classified based on their objectives, which may involve modifying molecules that are transported by extracellular vesicles or altering the targeting and interaction capabilities of extracellular vesicles within their surroundings. The most common techniques used for editing EVs, summarized in Figure 2, are detailed in the following paragraphs.

### 5.1. Loading of Cargoes

#### 5.1.1. Loading of Cargoes by Passive Loading

The simplest and most practical approach for loading cargoes into EVs involves their incubation. This method relies on transport mechanisms through passive means, which utilize concentration gradients and subsequent passive diffusion to facilitate the spontaneous incorporation of cargos into EVs or the cells secreting them [63]. Although the efficacy of this method is often inadequate, its loading efficiency can be impacted by factors such as the polarity of the cargoes [64]. Other factors influencing the process include temperature, time of contact with solvents [37], pH, and the concentration of the compounds to be loaded [56]. Notably, this way of loading generally should not have any impact on the structural or functional properties of the EVs.

Among other loading strategies by passive means, methods manipulating the pH with effect on the internal pH of exosomes, which is approximately 9, are also employed. Establishing a pH gradient that modifies the standard internal pH of the EVs to a pH of near 5 can enhance the efficiency of loading by up to threefold [65].

Hypotonic dialysis corresponds to another variant, wherein cargo and EVs are mixed within a dialysis membrane or tube [66]. The aforementioned process harnesses the differential concentration gradient to produce EVs laden with cargo. Compared to the employment of passive techniques alone, the utilization of hypotonic dialysis enhances the efficiency of EV loading by more than tenfold [67].

#### 5.1.2. Loading of Cargoes by Active Loading

##### Methods Underlying Physic Manipulation

Passive methods of loading may involve the application of recommended temperatures below minus 80 degrees Celsius, followed by thawing at room temperature in a cyclical fashion [68]. The abrupt alteration in temperature may slightly impede the integrity of membranes, enabling the incorporation of small molecules. Although this mechanism is relatively uncomplicated, it can lead to the fusion of extracellular vesicles (EVs) and has proven effective in merging EVs with liposomes [69,70]. Nonetheless, its encapsulation efficiency is generally moderate and often falls short of other physical methods like electroporating or sonicating the preparations [51].

Electroporation entails the application of short, yet intense electric pulses, resulting in the transient disruption of the cell membrane’s integrity. The process generates pores within the membrane, allowing hydrophilic compounds to penetrate and be enclosed within extracellular vesicles [71]. Frequently employed for loading EVs, the applied potential in electroporation can vary widely, ranging from 0.1 to 1000 kV, depending on the particular application. This method offers substantial loading efficiency and operational simplicity [57], it may also compromise the structural homeostasis of the membrane [71].

Sonication utilizes sonic forces to temporarily disrupt the reconstitution of EV membranes, enabling the passage of cargo throughout the lipidic walls of the vesicles [72]. Although sonication is effective in delivering high yield [73], it may have a greater impact on the integrity of the vesicles in comparison to other methods [74].

Intrinsic to the extrusion method, as another active loading mean, is the passage of vesicles through narrow pores, which can result in the mechanical disruption and reassembly of extracellular vesicles (EVs). To achieve this, an extruder device is utilized, complete with a heating block and polycarbonate filters that have precise pore sizes, typically spanning between 100 and 400 nanometers. The introduction of cargo into EVs is accomplished by continuously pushing the vesicle–cargo mixture through these filters [75]. Although this technique offers considerable packing efficiency and guarantees a uniform distribution of EV size [76], it may damage the vesicles, including their membranes and affect the signaling molecules in the membrane walls [67].

##### Methods Underlying Chemical Loading Assistance

Surfactant therapy typically entails the application of agents such as saponin or Triton, which induce pore formation in the membranes of EVs or cells. This results in increased permeability, enabling the passage of cargo and ultimately leading to significantly improved loading rates [77].

Similarly, the use of transfection entails facilitating cargo loading into EVs through the use of vectors. These vectors may include calcium phosphate [78], diethylaminoethyl-dextran [79], polyethyleneimine [80], or cell-penetrating proteins [81]. Moreover, structures like liposomes can be employed to introduce bigger cargoes, using the CRISPR/Cas9 system, through fusion with EVs [82]. However, it is necessary to acknowledge that particular vectors may possess the potential to inflict harm upon the target preparations [65].

### 5.2. Molecular Editing of the EVs Structure for Targeted Delivery

#### 5.2.1. Molecular Editing of EV Surfaces

There are various surface engineering strategies that can enhance the capability of EVs to transport cargo to designated locations. These techniques involve attaching particular molecules to the EV membrane using covalent or non-covalent bonds, without impairing the membrane’s integrity [83]. Peptides, proteins, or polymers can be attached to EV membranes by forming stable complexes [51]. Nonetheless, the use of toxic chemicals may be required for these applications, and these methods may necessitate careful consideration when applied to therapeutic EV editing [84]. Additionally, further purification steps are often required [85]. Non-covalent binding offers an alternative approach for modifying EV membranes in a stable manner [86]. One such strategy involves multivalent electrostatic interactions to facilitate membrane trespassing resulting from bioelectric interactions [87]. Nonetheless, it is crucial to note that the cationic nanomaterials used in this process may induce cytotoxicity through membrane disruption [88].

#### 5.2.2. Other Methods for the Modification of EV Surfaces

Further methods for editing the EV surface involve hybridization with liposomes. This method has been employed to incorporate larger cargoes into EVs [89] and to enhance nanovector delivery and the cellular uptaking of target cargoes [84].

## 6. Conclusions

The content of this review highlights the remarkable potential of bacterial, yeast, and plant EVs as candidates for the next generation of nanotransporters in the fields of biotechnology and biomedicine. Their extensive range of potential applications primarily revolves around the enhancement of drug delivery to the CNS or poorly vascularized skeletal tissues. Consequently, their anticipated impact on present and future advancements in the field is anticipated to be transformative and revolutionary.

EVs have demonstrated remarkable potential as advanced nanocarriers for delivering a wide range of substances, offering broad applications in both biotechnology and biomedicine. BP-EVs, which possess an exosomal and small-vesicle-related nature, exhibit exceptional compatibility for compound delivery in biological settings. Furthermore, they are capable of effectively navigating biological barriers and providing significant oral bioavailability. While physical methods can be used to manipulate BP-EVs, alternative strategies can also be employed to significantly modify their external and internal composition.

Despite the advantages that these novel sources of EVs present as potential nanocarriers for drug delivery and other applications, they also come with certain drawbacks and challenges. Although generally considered safe and biocompatible, these EVs, like other EV types, may exhibit heterogeneity in terms of size, cargo content, and surface properties. This diversity can complicate their characterization and standardization for specific applications. Finally, it is crucial to recognize that, like any novel therapeutic or drug delivery system, these vesicles, as nanovectors, may face regulatory hurdles and require thorough testing and approval before their promising clinical implementation.
